# Pathogenic variants in human DNA damage repair genes mostly arose after the latest human out-of-Africa migration

**DOI:** 10.3389/fgene.2024.1408952

**Published:** 2024-06-14

**Authors:** Jun He, Si Hoi Kou, Jiaheng Li, Xiaofan Ding, San Ming Wang

**Affiliations:** Department of Public Health and Medical Administration, Faculty of Health Sciences, Ministry of Education Frontiers Science Center for Precision Oncology, Cancer Centre and Institute of Translational Medicine, University of Macau, Taipa, China

**Keywords:** DDR genes, pathogenic variants, evolution origin, *TP53*, *BRCA*

## Abstract

**Introduction:**

The DNA damage repair (DDR) system in human genome is pivotal in maintaining genomic integrity. Pathogenic variation (PV) in DDR genes impairs their function, leading to genome instability and increased susceptibility to diseases, especially cancer. Understanding the evolution origin and arising time of DDR PV is crucial for comprehending disease susceptibility in modern humans.

**Methods:**

We used big data approach to identify the PVs in DDR genes in modern humans. We mined multiple genomic databases derived from 251,214 modern humans of African and non-Africans. We compared the DDR PVs between African and non-African. We also mined the DDR PVs in the genomic data derived from 5,031 ancient humans. We used the DDR PVs from ancient humans as the intermediate to further the DDR PVs between African and non-African.

**Results and discussion:**

We identified 1,060 single-base DDR PVs across 77 DDR genes in modern humans of African and non-African. Direct comparison of the DDR PVs between African and non-African showed that 82.1% of the non-African PVs were not present in African. We further identified 397 single-base DDR PVs in 56 DDR genes in the 5,031 ancient humans dated between 45,045 and 100 years before present (BP) lived in Eurasian continent therefore the descendants of the latest out-of-Africa human migrants occurred 50,000–60,000 years ago. By referring to the ancient DDR PVs, we observed that 276 of the 397 (70.3%) ancient DDR PVs were exclusive in non-African, 106 (26.7%) were shared between non-African and African, and only 15 (3.8%) were exclusive in African. We further validated the distribution pattern by testing the PVs in *BRCA* and *TP53*, two of the important genes in genome stability maintenance, in African, non-African, and Ancient humans. Our study revealed that DDR PVs in modern humans mostly emerged after the latest out-of-Africa migration. The data provides a foundation to understand the evolutionary basis of disease susceptibility, in particular cancer, in modern humans.

## 1 Introduction

A genome is constantly damaged by endogenous and exogenous factors (Liu and Huang, 2014; Bian et al., 2019). Effectively repairing the damaged DNA is vital to all life (Jeggo et al., 2016). The DNA damage repair (DDR) system is composed of multiple functional pathways and is universally preserved in living organisms. Each pathway consists of multiple genes that repair special types of DNA damage: BER (base excision repair) pathway repairs the lesions with small bases (Willey et al., 2014); DR (direct reversal) pathway repairs the DNA damaged by ubiquitous alkylating agents (Volkert, 1988); FA (Fanconi anemia) pathway repairs the damages of strand cross-link errors (Ceccaldi et al., 2016); HR (homologous recombination) (Liang et al., 2005) and NHEJ (nonhomologous end joining) pathways (Budman and Chu, 2005) repair double strand DNA breaks; MMR (mismatch repair) pathway repairs mismatched errors (Lynch et al., 2015); NER (nucleotide excision repair) pathway repairs the DNA damaged by helix-distorting DNA errors (Reardon and Sancer, 2006).

Genetic variation of insertion, deletion, duplication, indel, single nucleotide change etc. is common in DDR genes (Seplyarskiy and Sunyaev, 2021). While most genetic variants in DDR genes are benign or neutral, a portion of the variants are pathogenic that they damage the function of the affected DDR genes, causing genome instability and high risk of diseases. In medical terminology, a genetic variant can be classified into pathogenic, likely pathogenic, variant of uncertain significance, likely benign, or benign. Pathogenic and likely pathogenic variants (PVs) have direct clinical significance in disease diagnosis, treatment and prognosis but benign and likely benign variants (BVs) don’t, whereas the function of uncertain significance variants (VUS) remains unclear (Richards et al., 2015).

The PVs in DDR genes are well determined as the genetic predisposition for high disease risk, in particular, cancer (Lynch, 2010; Xue, et al., 2012; Fu, et al., 2014; Simons, et al., 2014). For example, the tumor suppressor *BRCA1* in the homologous DNA damage repair pathway plays essential roles in repairing double-strand DNA breaks. PVs in *BRCA1* lead to deficiency of the homologous DNA damage repair pathway, increasing the risk of breast and ovarian cancer (Welcsh and King, 2001; Narod and Foulkes, 2004). One of the distinct features of modern humans from many other mammals is the high susceptibility to cancer (David and Zimmerman, 2010). This could also be attributed to specific genetic factors in humans, to which the DDR PVs can be directly relevant. Determining the evolutionary origin and arising time of DDR PVs in modern humans can provide deep in-sight into the evolutionary basis of human diseases and develop strategies to control disease risk. Our recent studies in the human DDR genes including *BRCA1*, *BRCA2*, *TP53*, *MUTHY*, and *PALB2* etc. revealed that the DDR PVs were not transmitted from nonhuman species through cross-species conservation but arose in humans themselves (Li, et al., 2022; Chian, et al., 2023; Kou, et al., 2023; Xiao, et al., 2023). The results promoted us to question whether the result observed in these genes could be universal that the PVs in all human DDR genes were originated from humans themselves. We expanded the study by testing the origins of PVs in all 169 human DDR genes. The results confirmed that nearly all PVs in the 169 DDR genes were originated in humans themselves (Zhao, et al., 2024). However, human evolution has lasted for over 6 million-years since the separation from Chimp. While our study determined that the PVs in human DDR genes is basically originated from humans themselves, the precise time point for when the PVs arose along human evolutionary history remain unknown. One of the most significant events in human evolution history is the latest out-of-Africa migration, which played fundamental roles in the formation of modern humans. Our previous study (Zhao et al., 2024) used the PV data from ancient humans determine that the origin of human DDR PVs was from humans themselves. However, the precise arising timing of the DDR PVs along human evolution process remains to be determined.

Modern non-African humans (hereafter: non-African) were the direct descendants of the latest out-of-Africa human migrants that occurred 50,000–60,000 years ago (Hublin, et al., 2017) staying largely in Persian Plateau and spread to the world 45,000 years ago (Vallini et al., 2024). The non-African migrants were more homogeneous than the ancestral African due to the founder effects and the bottleneck effects of small population size when out-of-Africa migration occurred (Tishkoff, et al., 2009). Although out-of-Africa humans could inherit certain genetic variants from their African ancestors, novel genetic variation should arise and be selected in the decedent out-of-Africa humans following their adaptation to new environments and increased population size. These novel variants should be largely absent in the ancestors of the out-of-Africa humans and the descendants of ancient African (hereafter: African) except these occurred coincidentally. Rapid progress in archaeological genomics has led to the identification of rich genetic variation data including the PVs from ancient humans, which were mostly from the descendants of out-of-Africa migrants composed of the ancient non-African living in Eurasia continent within the last 10,000 years (Kou, et al., 2023). The main goal of our current study is to narrow down the arising time of human DDR PVs by referring to the out-of-Africa migration as the cutting point. We consider that the assumptions “zoom-in” from the million-year human evolution history to the Out-Of-Africa period of 60,000 years ago, which played one of the most significant roles in the formation of modern humans, to determine the arising time of human DDR PVs. The results from the much simplified analytic process can open a door to further study the complexities of DDR PV origins. Thus, we hypothesized that comparison of the DDR PVs among African, non-African and ancient humans should allow to determine the arising time by referring out-of-Africa migration as the cutting point. The PVs absence in African but sharing between ancient humans and non-African would imply that the PVs were originated after the out-of-Africa migration; the PVs shared in African, ancient humans and non-African would imply that the PVs were originated in African; and the PVs present only in African but not in ancient humans and non-African would imply that the PVs were originated in African.

To test our hypotheses, we identified the DDR PVs in 169 human DDR genes originated in 28,872 African, 222,342 non-African, and 5,031 ancient humans. We made direct comparison of DDR PVs between African and non-African. We also used the DDR PVs of ancient humans as the intermediate to further compare the DDR PVs between African and non-African. We further validated the results by comparing the PVs in *BRCA* and *TP53* in the three cohorts. The results reveal that DDR PVs in modern humans mostly arose after the latest human out-of-Africa migration.

## 2 Materials and methods

### 2.1 Sources of human DDR PVs

The following databases were used to extract African and non-African PV data: gnomAD combing v2 (125,748 exomes) and v3 (76,156 genomes); only the noncancer data were included in the study (https://gnomad.broadinstitute.org/) (Karczewski, et al., 2020); jMorp (https://jmorp.megabank.tohoku.ac.jp/, genome variation data, v202ƒ (20,630, 38,722 samples) (Tadaka, et al., 2019); and ChinaMAP (http://www.mbiobank.com/, v2020-03.beta, 10,588 samples) (Cao, et al., 2020). Each dataset (vcf files) was annotated against the coding regions of DDR genes by ANNOVAR (https://annovar.openbioinformatics.org/en/latest/) (Wang, et al., 2010), using hg38 as the reference. The 169 DDR genes were identified in our previous work (Qin, et al., 2022) by referring to “Replication and Repair” in the KEGG PATHWAY database (https://www.genome.jp/kegg/pathway.html#genetic) (Kanehisa and Goto, 2000) and the Human DNA Repair Genes database (https://www.mdanderson.org/documents/Labs/Wood-Laboratory/human-dna-repair-genes.html) (Wood, et al., 2005). The PVs included the Pathogenic and Likely Pathogenic classified by ClinVar (released on 20 March 2022) (Landrum, et al., 2016). The PVs located in coding exons were used for the analyses. Based on the origins of the extracted PVs, the PVs from different databases were combined to form the African PV and non-African PV datasets. Only single-base PVs were included in the study.

### 2.2 Ancient human genomic data analysis

Ancient human genome sequences were collected from “Allen Ancient DNA Resource” (version 50.0, https://reich.hms.harvard.edu/allen-ancient-dna-resource-aadr-downloadable-genotypes-present-day-and-ancient-dna-data, accessed 10 October 2021) and its listed publications. A total of 5,031 ancient individual genomic datasets dated between 45,045 and 100 years before present (BP) were included in the study. MapDamage 2.0 (version 2.1.1) (Jonsson, et al., 2013), a base recalibration tool used to remove false variants generated by deamination of ancient DNA, was used to check the quality of the data. SAMtools (Li, et al., 2009) was used to call variants to generate a VCF file (http://www.htslib.org/). The called variants were further processed by the ANNOVAR program (https://ANNOVAR.openbioinformatics.org/en/latest/) (Wang, et al., 2010). The annotated variants were compared with ClinVar to obtain related information for those matched by the ancient variants. The locations of ancient PV carrier’s fossil excavations and the estimated timing were based on the original data sources and publications.

### 2.3 Statistical analysis

Z-test, T-test, regression analysis, and phylogenetic clustering were performed to characterize the population differences using R, and *p* < 0.05 was set as the significant level if applicable. Geographical map of the matched ancient PV carriers was generated by using MATLAB version R2022a (The MathWorks, Inc.).

## 3 Results

**FIGURE 1:**
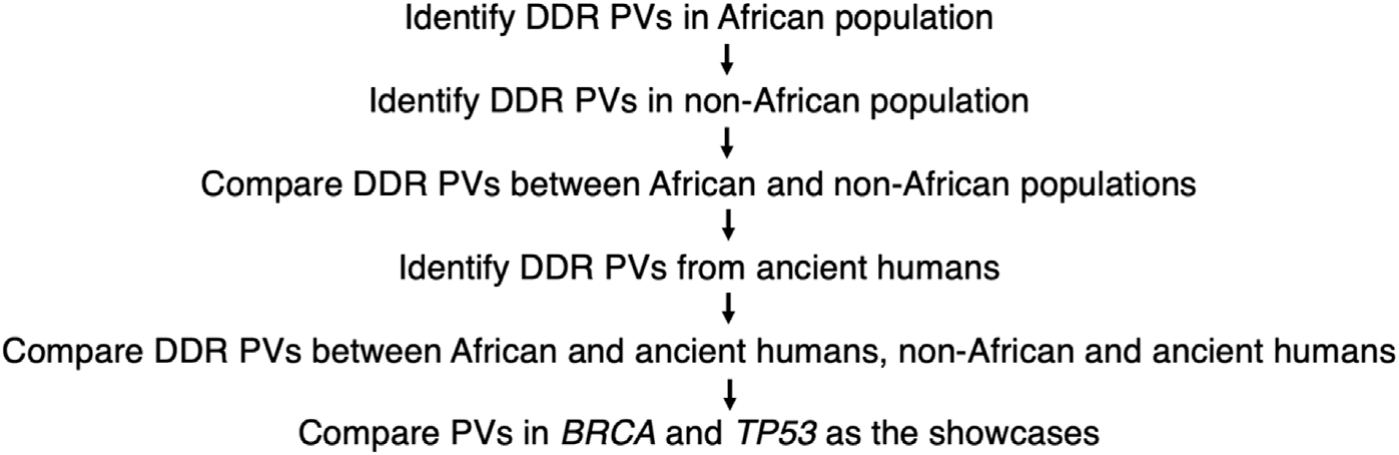
Scheme of the analytic process. See text for detailed explanation.

We designed the strategy for our study: 1. Direct comparing the DDR PVs between African and non-African populations to estimate the similarity and difference of DDR PVs between the two populations after their separation since out-of-Africa migration; 2. Using the DDR PVs derived from ancient humans as the reference to further test the similarity and difference of DDR PVs between the two populations; 3. Using the PVs in *BRCA* and *TP53* as the examples to validate the results from the comparisons. Figure 1 outlines the working flow of analytic process.

### 3.1 DDR PVs identified in African and non-African

Modern non-African humans (hereafter: non-African) were the direct descendants of the latest out-of-Africa human migrants that occurred 50,000–60,000 years ago (Hublin, et al., 2017). In addition to the genetic variation inherited from the African ancestors, *de novo* variation including DDR PVs can arise in non-African in reflecting their adaptation to new global environments. The first step of our study was to collect DDR PVs from African and non-African for further comparison.

We mined DDR PVs from the genomic data covering 251,214 individuals, including 28,872 (11.5%) African individuals and 222,342 (88.5%) non-African individuals. Table 1 lists the compositions of non-African population. We identified a total of 1,060 single-base DDR PVs in 77 human DDR genes, distributed in 8 DDR pathways of base excision repair (BER), DNA damage response (DNA res), DNA replication (DNA rep), Fanconi anemia (FA), homologous recombination (HR), mismatch repair (MMR), nonhomologous end joining (NHEJ), and nucleotide excision repair (NER) (Table 2; Supplementary Table S1). At the pathway level, the FA pathway had the highest number of 493 PVs among the 49 DDR genes in the pathway (Table 2A); at the gene level, *ATM* had the highest number of 104 PVs among the 77 PV-containing DDR genes. Of the 1,060 PVs, 378 (35.7%) were found in the *ATM*, *BRCA1, BRCA2, FANCA,* and *MUTYH* genes (Table 2B), and stop-gain PV was the major variation type contributing to more than 70% of all PVs (Table 2C).

**TABLE 1:** Composition of non-African populations.

Non-African population	Cases
Non-Finnish European	90,914
East Asian	61,111
Latino/Admixed American	24,943
South Asian	17,727
Finnish	16,140
Ashkenazi Jewish	6,776
Other (population not assigned)	4,117
Amish	456
Middle Eastern	158
Total	222,342

**TABLE 2:** Germline DDR PVs identified in Africans and non-Africans.

A. DDR pathways and genes with PVs			
Pathways	No. genes	Gene with PVs (%)	No. PVs
Homologous Recombination	37	21 (56.8)	472
DNA damage response	15	4 (26.7)	66
Fanconi anemia	49	30 (61.2)	493
Mismatch Repair	20	9 (45.0)	134
Nonhomologous end joining	13	6 (46.2)	72
Nucleotide excision repair	41	15 (36.6)	121
Base excision repair	32	8 (25.0)	63
DNA replication	34	12 (35.3)	35
Total	166	77 (45.6)	1,060

B. DDR genes with PVs			
Gene	No. PVs	Gene	No. PVs
ATM	104	RAD54L	6
BRCA2	92	ERCC1	5
BRCA1	72	FANCE	5
FANCA	65	RNASEH2A	5
MUTYH	45	RNASEH2C	5
PMS2	39	XRCC4	5
RAD50	37	DDB2	4
TP53	37	DNA2	4
MSH6	35	FANCL	4
PALB2	35	NHEJ1	4
ERCC6	26	RBBP8	4
BARD1	25	SLX4	4
CHEK2	25	ATR	3
MSH3	25	MCM7	3
BRIP1	22	RNASEH1	3
ERCC2	22	TOP3A	3
BLM	19	FAN1	2
MLH1	19	FANCF	2
FANCC	17	LIG1	2
FANCD2	16	LIG3	2
XPC	15	MCM2	2
RAD51C	14	POLK	2
FANCI	12	TELO2	2
MRE11	12	UNG	2
NBN	12	GTF2H5	1
ERCC8	11	MCM4	1
FANCM	11	MLH3	1
MSH2	11	PCNA	1
ERCC3	10	POLE	1
FANCG	9	RAD51	1
NTHL1	9	RAD51B	1
RAD51D	9	RAD54B	1
BIVM-ERCC5	8	REV3L	1
LIG4	8	RPA1	1
POLH	8	SYCP3	1
ERCC4	7	UBE2T	1
RNASEH2B	7	XRCC1	1
XPA	7	XRCC2	1
DCLRE1C	6		
Total			1,060

C. Types of PV variation			
Variation type		Number of PVs	Rate (%)
Stop gained		747	70.5
Missense		271	25.6
Start lost		24	2.3
Synonymous		17	1.6
Stop loss		1	0.1
Total		1,060	100

**FIGURE 2:**
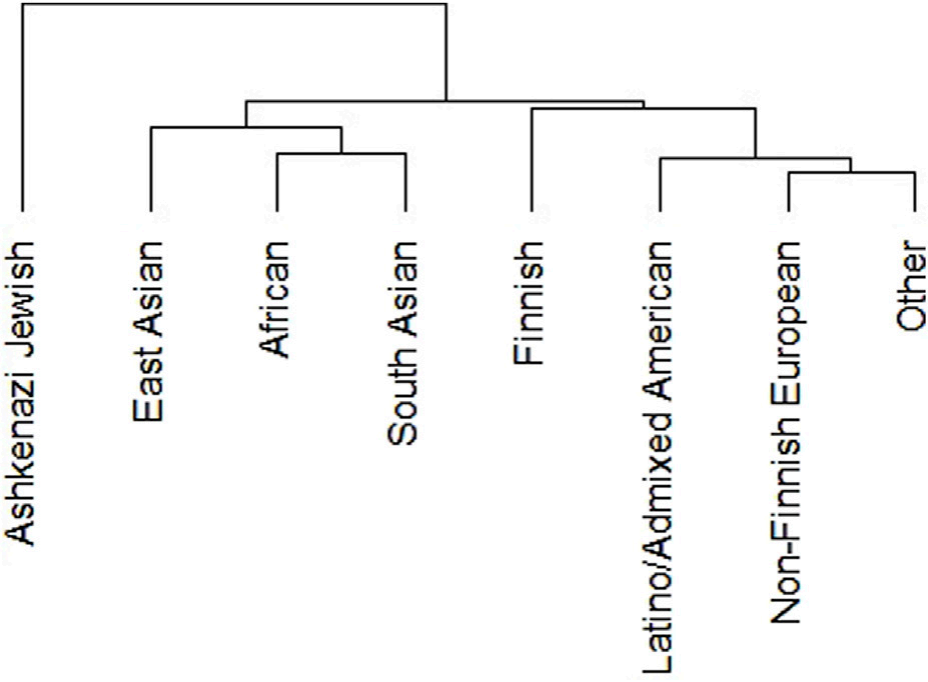
Clustering of DDR PVs in different ethnic groups of non-African population. It shows that DDR PVs are clustered following the ethnic relationship between different ethnic groups.

We performed phylogenetic test to know the relationship of DDR PVs among ethnic groups in non-African population. We removed Amish and middle-eastern groups from the analysis due to the small size of these groups (less than 1,000). The results show indeed DDR PV follows the ethnic relationship between different populations (Figure 2).

The DDR PVs were identified from different ethnic populations. We compared the PVs in non-Finnish European group with 620 PVs and East Asian group with 313 PVs. The results showed that only 140 PVs were shared between the two groups, accounting for 23% of European PVs and 45% of East Asian PVs, whereas the rest PVs were only present in each group. The results highlighted the ethnic-specific nature of DDR PVs. Further, the PVs were rare variants with low population frequency, supporting their pathogenicity (Supplementary Table S2).

### 3.2 Direct comparison of DDR PVs between African and non-African

**FIGURE 3:**
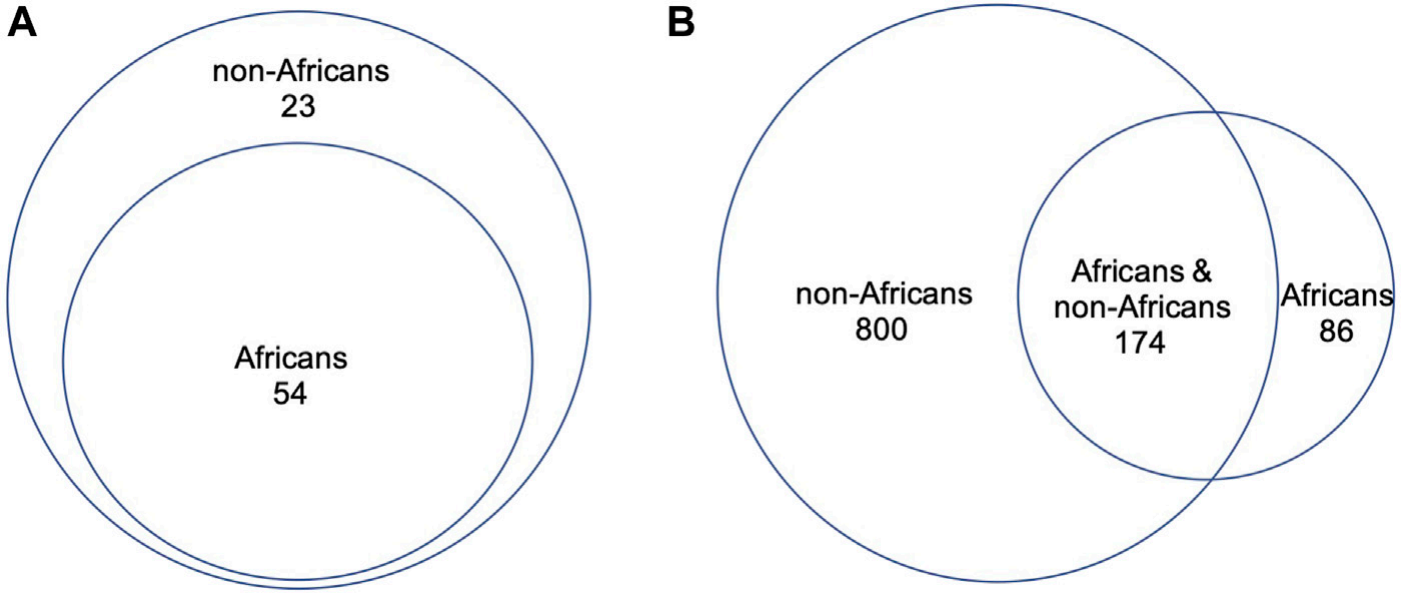
Direct comparison of DDR PVs between African and non-African. **(A)**. PV-affected DDR genes between African and non-African. It shows that all the PV-affected DDR genes in African were included in the PV-affected DDR genes in non-African; **(B)**. DDR PVs between African and non-African. It shows that most of the DDR PVs in non-African were not present in African.

**FIGURE 4:**
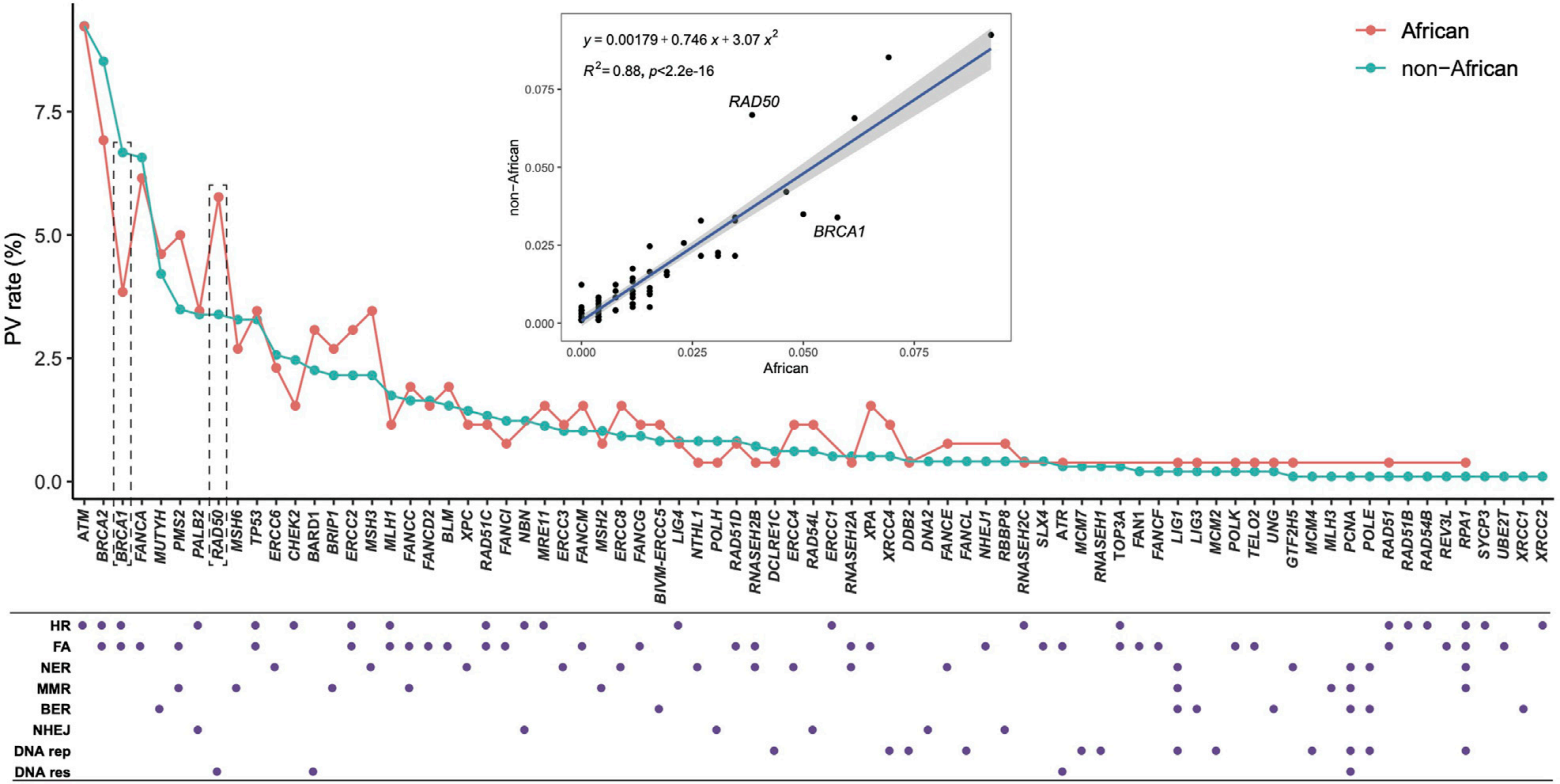
Quantitative distribution of DDR PVs, affected DDR genes and pathways between African and non-African. It shows that the patterns of the quantitative distribution of DDR PVs were highly correlated between African and non-African, except for *BRCA1* and *RAD50*. HR, homologous recombination; FA, Fanconi anemia; NER, nucleotide excision repair; MMR, mismatch repair; BER, base excision repair; NHEJ, nonhomologous end joining; DNA rep, DNA replication; DNA res, DNA damage response.

We compared directly the DDR PVs between African and non-African. The results showed 1). Higher carrier rate of DDR PV in African than in non-African. Of the 1,060 PVs, 260 PVs among the 54 DDR genes were in 618 African individuals (4.2 PVs/individual on average), and 974 PVs in 77 DDR genes were in 7,691 non-African individuals (1.3 PVs/individual on average) (Supplementary Table S1). The high carrier rate of DDR PV in African compared with non-African was consistent with the high diversity of African genome among different ethnic human populations (Tishkoff, et al., 2009); 2). The DDR PVs affected the same DDR pathways and genes between African and non-African. At the pathway level, all eight DDR pathways in both African and non-African carried DDR PVs (Table 2; Supplementary Table S1); at the gene level, there were 54 PV-containing DDR genes in African and 77 in non-African, all 54 PV-containing DDR genes in African were included in the 77 PV-containing DDR genes in non-African (Figure 3A); 3). Similar prevalence of DDR PVs between African and non-African. The prevalence of DDR PVs was highly correlated between African and non-African, except for the PVs in a few DDR genes. For example, *BRCA1* PVs were lower in African than in non-African and *RAD50* PVs were higher in non-African than in African (Figure 4); 4). Most DDR PVs were not shared between African and non-African. 86 (33.1%) of the 260 PVs in African and 800 (82.1%) of the 974 PVs in non-African were not shared (Figure 3B; Supplementary Table S3). For the 174 DDR PVs shared between African and non-African, the highly prevalent PVs in 5 DDR genes of *MUTYH* p.Gly368Asp, *MUTYH* p.Tyr151Cys, ERCC3 p.Arg452Ter, ERCC4 p.Trp193Ter, and RNASEH2B p.Ala177Thr contributed 30% (141 out of 471) of the total PV carrier, and 4 *TP53* PVs of c.473G>A p.Arg158His, c.733G>A p.Gly245Ser, c.743G>A p.Arg248Gln, c.818G>A p.Arg273His were all *TP53* hot-spot PVs (Baugh et al., 2018), and the European founder mutations of MUTYH c.452A>G p.Tyr151Cys and c.1103G>A p.Gly368Asp (p.Tyr179Cys and p.Gly396Asp) were represent in African (Casper, et al., 2012) (Supplementary Table S4).

We applied Z test for Figure 3 to test the significant difference between the ratios of the involved genes to all the 169 DDR genes (*p* = 0.01404), and for Figure 4 to show the significant difference between the ratios of the variants to the respective population sizes (*p* < 2.2e-16). We also performed T-test for the significant difference of DDR PV carriers between African and non-African populations (*p*-value = 0.00712 between the last two columns in Supplementary Table S1).

The results indicated that the spectrum of DDR PVs between African and non-African were substantially different.

### 3.3 Comparison of ancient DDR PVs in African and non-African

Recent progress in archaeological genomics has generated rich genomic sequence data from ancient humans distributed in the Eurasia continent. We collected genomic data from 5,031 ancient humans covering most ancient human genomic data publicly available to date. Although the dated time for the cases spans between 45,045 and 100 years, nearly 80% were within the last 6,000 years but only 2.5% were older than 10,000 years (Table 3; Supplementary Table S5). Therefore, the ancient samples included in our study were mostly within the last 6,000 years of the descendants of out-of-Africa migrants, reflecting the human population expanded following agricultural revolution. The narrow timing window of the samples used in the study minimized the impact of possible variation differences along longer period on data consistency.

**TABLE 3:** Dated timing of ancient human samples used in the study.

Timing (year before present)	Number of cases	Rate (%)
10,000–37470	128	2.5
8,000–9,999	133	2.6
6,000–7,999	579	11.5
4,000–5,999	969	19.3
2000–3,999	2,002	39.8
<2000	928	18.4
NA	292	5.8
Total	5,031	100

The DDR PVs from the ancient humans can be used as a valuable intermediator to further test the relationship of DDR PVs between African and non-African. High sharing between ancient human, African and non-African would indicate the high likelihood that the DDR PVs occurred before the out-of-Africa migration therefore arose in African before out-of-Africa migration; and high sharing between ancient humans and non-African but not African would indicate the high likelihood that the DDR PVs arose after the out-of-Africa migration.

We first identified 930 PVs in 70 DDR genes from 5,031 ancient humans, which were dated between 37,470 and 190 years BP, all were from the fossils in Eurasia continent except two cases from Cameroon (sample ERR3607244 dated 3,105 years BP carrying ERCC1 c.121C>T p.Arg41*, sample ERR3607243 dated 3,065 years BP carrying MUTYH c.1180C>T p.Gln394*) (Supplementary Table S6).

**FIGURE 5:**
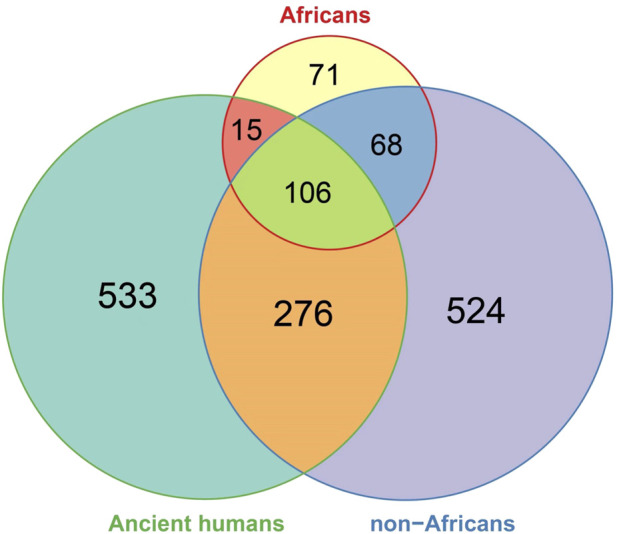
Comparison of DDR PVs between ancient humans, African, and non-African. It shows that of the shared DDR PVs, those shared between ancient humans and non-African were more common than those shared between ancient humans and African, between African and non-African, and among all the three groups.

We then compared the ancient DDR PVs with the DDR PVs in African and non-African. Of the 930 ancient DDR PVs, 397 in 56 DDR genes were shared and 533 were not shared with African or non-African (Supplementary Table S7). We focused on the shared PVs for further comparison. The 397 shared ancient PVs were derived from 964 ancient humans dated between 30,375 and 190 years BP (Supplementary Table S7). We observed 3 types of sharing pattern: 1). 15 (3.8%) PVs were shared with African only; 2). 106 (26.7%) PVs were shared with both African and non-African, and 3). 276 (69.5%) PVs were shared with non-African only (Figure 5; Supplementary Table S7). The results indicate that the ancient DDR PVs were mostly present in non-African.

### 3.4 Comparison of *TP53* and *BRCA* PVs between ancient humans, African and non-African

**FIGURE 6:**
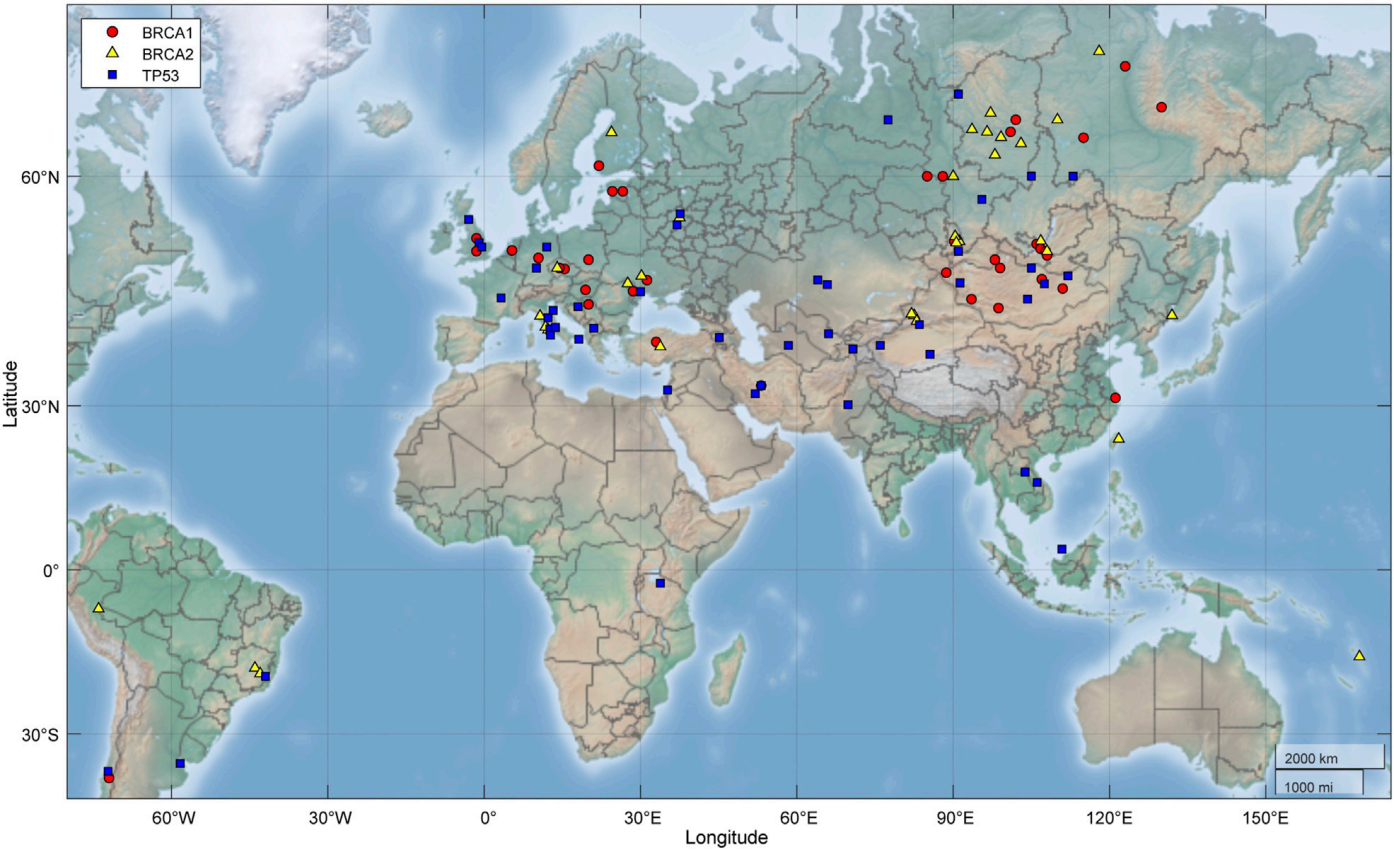
Geographic locations of ancient fossils carrying *BRCA* and *TP53* PVs. It shows the PV carriers distributed across the Eurasia continent. Red: Fossils carrying *BRCA1* PVs; yellow: Fossils carrying *BRCA2* PVs; blue: Fossils carrying *TP53* PVs.

**FIGURE 7:**
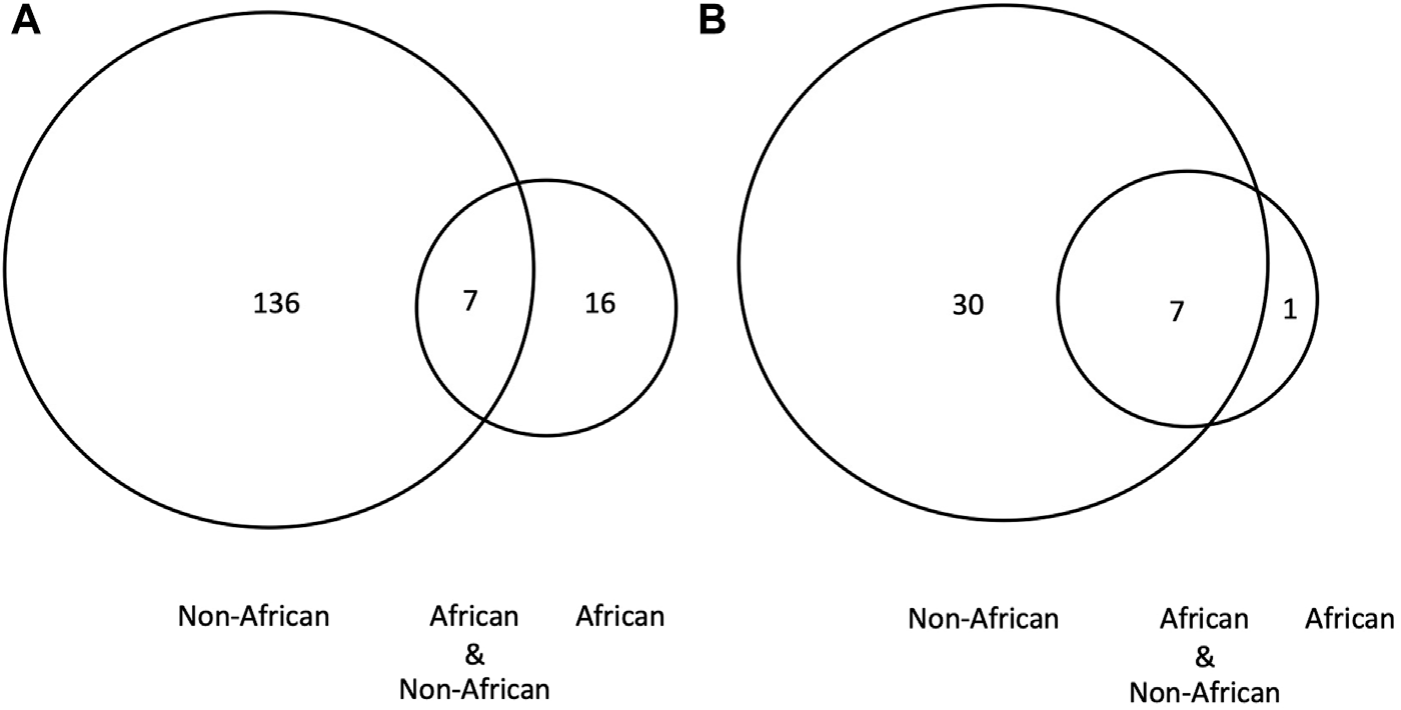
*BRCA* PVs between African, non-African and ancient humans. **(A)**. Direct comparison of *BRCA* PVs between African and non-African. It shows that of the 143 *BRCA* PVs in non-African, 136 were not shared with African; **(B)**. Comparison of *BRCA* PVs between African, non-African, and ancient humans. It shows that of the 38 PVs in ancient humans, 30 were not shared with African. The results demonstrated that the *BRCA* PVs in non-African were largely different from African regardless the absence or presence of ancient *BRCA* PVs.

We used the *TP53* and *BRCA* (*BRCA1*/*BRCA2*) PVs as examples to further illustrate the relationship of the DDR PVs between ancient humans, non-African and African. We observed the following results from the analysis:

1) *TP53* PVs. Of the 397 ancient DDR PVs shared between African and non-African, there were 19 ancient *TP53* PVs. Of the 19 PVs, only 2 (10.5%) were shared with African only; 4 (21%) were shared with both African and non-African, all were TP53 hotspot PVs (c.473G>A p.Arg158His, c.733G>A p.Gly245Ser, c.743G>A p.Arg248Gln, c.818G>A p.Arg273His); and 13 (68%) were shared with non-African only, 3 were TP53 hotspot PVs. The PV carriers of non-African were dated between 34,425 and 2,409 BP (Table 4), and were distributed in different geographic locations across the Eurasia continent (Figure 6; Supplementary Table S8).
2) *BRCA* PVs. Direct comparison of *BRCA* PVs between African and non-African showed that of the 143 *BRCA* PVs in non-African, 136 (95.1%) were not shared with African (Figure 7A). Using Ancient human *BRCA* PVs as the intermediate identified 38 *BRCA* PVs dated between 37,470 and 419 years BP, of which only 1 (2.6%) was present in African, 7 (18.4%) in both African and non-African, and 30 (78.9%) in non-African only (Figure 7B; Supplementary Table S9). The non-African carriers were dated across a wide range between 37,470 and 419 years BP (Table 4), and spread mainly in the Eurasia continent (Figure 6).

**TABLE 4:** Ancient *TP53* and *BRCA* PVs shared with Africans and non-Africans.

cDNA	Protein	Type of variation	Shared with		Earliest time (BP)
			African	Non-African	
*TP53*					
c.814G>A	p.Val272Met	nonsynonymous SNV	-	+	2,409
c.1010G>A	p.Arg337His	nonsynonymous SNV	-	+	2,530
c.638G>A	p.Arg213Gln	nonsynonymous SNV	-	+	2,549
c.736A>G	p.Met246Val	nonsynonymous SNV	-	+	3,328
c.542G>A	p.Arg181His	nonsynonymous SNV	-	+	4,450
c.817C>T	p.Arg273Cys	nonsynonymous SNV	-	+	4,500
c.451C>T	p.Pro151Ser	nonsynonymous SNV	-	+	4,500
c.455C>T	p.Pro152Leu	nonsynonymous SNV	-	+	4,500
c.713G>A	p.Cys238Tyr	nonsynonymous SNV	-	+	4,725
c.853G>A	p.Glu285Lys	nonsynonymous SNV	-	+	6,713
c.844C>T	p.Arg282Trp	nonsynonymous SNV	-	+	6,960
c.742C>T	p.Arg248Trp	nonsynonymous SNV	-	+	10,000
c.734G>A	p.Gly245Asp	nonsynonymous SNV	-	+	34,425
c.473G>A	p.Arg158His	nonsynonymous SNV	+	+	4,160
c.818G>A	p.Arg273His	nonsynonymous SNV	+	+	6,415
c.733G>A	p.Gly245Ser	nonsynonymous SNV	+	+	6,483
c.743G>A	p.Arg248Gln	nonsynonymous SNV	+	+	7,515
c.841G>A	p.Asp281Asn	nonsynonymous SNV	+	-	1724
c.375G>A	p.Tyr126Valfs*23	synonymous SNV	+	-	2,927
*BRCA1*					
c.4573C>T	p.Gln1525*	stopgain	-	+	419
c.5497G>A	p.Val1833Met	nonsynonymous SNV	-	+	2,185
c.5096G>A	p.Arg1699Gln	nonsynonymous SNV	-	+	2,600
c.5444G>A	p.Trp 1815*	stopgain	-	+	4,079
c.514C>T	p.Gln172*	stopgain	-	+	4,500
c.962G>A	p.Trp321*	stopgain	-	+	4,500
c.5503C>T	p.Arg 1835*	stopgain	-	+	4,575
c.34C>T	p.Gln12*	stopgain	-	+	4,839
c.2338C>T	p.Gln780*	stopgain	-	+	5,350
c.3403C>T	p.Gln1135*	stopgain	-	+	6,319
c.4675G>A	p.Glu1559Argfs*15	nonsynonymous SNV	-	+	6,373
c.1687C>T	p.Gln563*	stopgain	-	+	6,483
c.4834C>T	p.Gln1612*	stopgain	-	+	7,200
c.5095C>T	p.Arg1699Trp	nonsynonymous SNV	-	+	9,872
c.181T>G	p.Cys61Gly	nonsynonymous SNV	-	+	37,470
c.4327C>T	p.Arg1443*	stopgain	+	+	4,600
*BRCA1*					
c.3607C>T	p.Arg1203*	stopgain	+	+	6,415
c.5251C>T	p.Arg1751*	stopgain	+	+	NA
*BRCA2*					
c.5992C>T	p.Gln 1998*	stopgain	-	+	450
c.3G>A	p.?	startloss	-	+	1964
c.5773C>T	p.Gln 1925*	stopgain	-	+	1964
c.7757G>A	p.Trp2586*	stopgain	-	+	3,000
c.8377G>A	p.Gly2793Arg	nonsynonymous SNV	-	+	3,070
c.8754G>A	p.=	synonymous SNV	-	+	3,532
c.9117G>A	p.=	synonymous SNV	-	+	4,225
c.2224C>T	p.Gln742*	stopgain	-	+	4,239
c.7615C>T	p.Gln2539*	stopgain	-	+	4,450
c.9154C>T	p.Arg3052Trp	nonsynonymous SNV	-	+	4,800
c.7480C>T	p.Arg2494*	stopgain	-	+	6,319
c.6952C>T	p.Arg2318*	stopgain	-	+	7,515
c.772C>T	p.Gln258*	stopgain	-	+	10,000
c.7878G>A	p.Trp2626*	stopgain	-	+	10,000
c.92G>A	p.Trp31*	stopgain	-	+	31,630
c.8009C>T	p.Ser2670Leu	nonsynonymous SNV	+	+	400
c.9294C>G	p.Tyr3098*	stopgain	+	+	2,221
c.7558C>T	p.Arg2520*	stopgain	+	+	2,305
c.9382C>T	p.Arg3128*	stopgain	+	+	8,610
c.7115C>G	p.Ser2372*	stopgain	+	-	1,595

We further used the *BRCA* PVs from modern African as an additional reference to test the relationship of *BRCA* PVs between African and non-African. The Consortium of Investigators of Modifiers of BRCA1/2 (CIMBA) identified 134 *BRCA* PVs (61 in *BRCA1* and 73 in *BRCA2*) from African and descendants (Friebel, et al., 2019). Comparison of the 134 African *BRCA* PVs with the 38 ancient *BRCA* PVs showed that only 2 PVs (*BRCA2* c.7115C>G and *BRCA2* c.8009C>T) were shared between the two datasets (Supplementary Table S9).

The results from the comparisons showed a consistent pattern that the majority of DDR PVs were present in non-African, a small part was shared between African and non-African, and only limited PVs were present only in African.

## 4 Discussion

Determination of the evolution origin and arising time of PVs in human DDR genes is important, as it will provide information to address the following fundamental questions in human genetics and human diseases: 1. Understand the genetic basis of human cancer. A fundamental question in biology is “where we come, where we go.” The same applies for genetic variation. Only after knowing the origin and arising time, we can better understand the genetic basis of human cancer risk. The germline PVs in human DDR genes provides an ideal system to address the issue; 2. Understand the origin of human disease susceptibility. The PVs in human DDR genes are well determined as the genetic predisposition in causing high risk of cancer by functioning as the first hit in initiating the oncogenic process. This is best represented by many “founder mutation” in human DDR genes, such as the 185delAG (c.68_69delAG) founder mutation in Ashkenazi Jews that causing high risk of breast cancer in Ashkenazi Jews population. Determination of the origin and timing of the mutation greatly enhances cancer prevention in the population; and 3. Understand why modern humans are at much higher cancer risk than many other mammals such as elephant, whales, and chimpanzee.

By systematically comparing DDR PVs among modern African, modern non-African, and ancient humans, our study reveals that most of the DDR PVs in modern humans originated after the latest out-of-Africa migration.

Our current study aims to understand the arising time of disease-causing DDR PVs risk in modern humans by referring the well-established Out-of-Africa model (Hublin, et al., 2017). The key points of the model include that 1). African is the most genetically diversified among global ethnic human populations; 2). Modern humans were originated from African; 3). Only part of genetic variants in the descendants of out-of-Africa migrants was inherited from African ancestors; 4). *de novo* genetic variation could arise in the descendants of out-of-Africa migrants by adaptation, selection and population expansion. The *de novo* genetic variants were absent in their African ancestors.

The increased population size since the latest out-of-Africa migration can be an important factor contributing to the *de novo* variants, as the increased population size can greatly increase the number of DDR PVs in the human population considering the consistent mutation rate in the human genome (Cabrera, 2021). It is determined that only a few hundred to thousands of individuals moved out of Africa 60,000 years ago (Zhivotovsky, et al., 2003). Since then, the size of human population had increased slowly until the end of the last glacial period approximately 10,000 years ago (Henn et al., 2012). Following the agricultural revolution occurred 6,000 years ago (Gignoux, et al., 2011) and the industrial revolution centuries ago, the size of human population has been expanding significantly till the current size of modern humans. Although the same DDR pathways and DDR genes were affected by DDR PVs in both African and non-African, the spectrum of DDR PVs between African and non-African differs substantially after their separation by the latest out-of-Africa migration. After about 2,000 generations since the latest out-of-Africa migration, *de novo* DDR PVs can arise constantly following the expansion of the human population (Hudjashov, et al., 2007). The high PV sharing rate between ancient humans and non-African but low between ancient humans and African is due to the relationship of ancient humans closer to non-African (within 10,000 years) than African. These findings also explain the high ethnic specificity of DDR PVs in modern humans (Bhaskaran et al., 2021; Qin et al., 2022; Qin et al., 2023). The fact that most human DDR PVs have only a few thousand years of history also implies that human DDR PVs could be too young to allow evolutionary selection to function effectively. Whether the PVs would be eliminated or integrated into human genome re-mains to be determined with longer time.

For the following reasons, *TP53* and *BRCA* were selected from all DDR genes as the examples for detailed analysis. As the most mutated tumor suppressor gene, *TP53* is mutated in more than half of human cancer types (Narod and Foulkes 2004). The presence of a group of hot-spot PVs in African makes *TP53* a valuable tool to test the origin of DDR PVs (Olivier et al., 2010; Monti et al., 2020). The finding that only 2 of the 19 ancient *TP53* PVs were shared with African is consistent with the observation that *TP53* variation has lower prevalence in African than in non-African (0.07% in African, 0.12% in South Asian, 0.15% in East Asian, 0.16% in Latin American, and 0.24%–0.28% in European) (de Andrade et al., 2017), whereas *TP53* hotspot PVs are also highly prevalent in modern humans (Freed-pastor and Prives, 2012; Baugh et al., 2018; Kou et al., 2023). As represented by the four *TP53* hotspot PVs, those shared between African and non-African were likely originated from African and inherited by non-African after the out-of-Africa migration. *BRCA* is under strong positive selection in humans (Huttley, et al., 2000; Fleming, et al., 2003; Abkevich, et al., 2004; Pavlicek, et al., 2004; Burk-Herrick, et al., 2006) for its new roles gained in regulating immunity (O’Connell, 2010), gene expression and metabolism (Lou, et al., 2014), neural development (Rosen, et al., 2006), and reproduction (Smith et al., 2013; Pao, et al., 2014). *BRCA* variation is also highly prevalent in non-African and African with high ethnic-specificity (Qin et al., 2022). These features make *BRCA* PVs as ideal markers to test the relationship of DDR PVs between African and non-African before and after out-of-Africa migration. Our previous studies revealed that the PVs in *TP53* and *BRCA* were originated from humans themselves but not from non-human species (Li et al., 2022; Kou et al., 2023). Furthermore, rich PV data in *TP53* and *BRCA* are available and widely applied in guiding clinical cancer diagnosis, treatment and prognosis (Cline et al., 2018; de Andrade et al., 2022). These features made *TP53* and *BRCA* as ideal models to validate the observed DDR PVs between African and non-African populations.

Direct comparison of the DDR PVs between African and non-African showed substantial differences (Figure 3). However, the results could be biased due to the different population sizes of African and non-African included in the study. We further used the DDR PVs from ancient humans as the intermediate for the comparison. The ancient humans used in the study were mostly dated within the last 10,000 years, therefore, closer to non-African than the African. Using the ancient DDR PVs for the comparison eased the uncertainty attributed by different population sizes and enhanced the reliability of the results.

The results from our study have an immediate impact on the characterization of human DDR PVs. Many genetic variants have been identified, but their pathogenicity remain to be determined. For example, 89.7% of the 72,330 *BRCA* variants identified in modern humans remain uncharacterized (https://brcaexchange.org/factsheet, accessed 8 December 2023). To ease the situation, *in silico* computational programs are often applied to predict pathogenicity for the unknown variants. Certain computational programs are designed using the concept of evolutionary conservation. However, our studies showed that DDR PVs did not originate from nonhuman species through evolutionary conservation but arose from humans themselves (Li, et al., 2022; Chian, et al., 2023; Kou, et al., 2023; Xiao, et al., 2023), and our current study further reveals that most human DDR PVs arose after the latest human out-of-Africa migration. While evolutionary conservation-based approaches can be powerful in analyzing the benign variants in DDR genes highly conserved between human and nonhuman species, they are not suitable in identifying the PVs in human DDR genes as they are basically absent in non-human species. Instead, focusing on the humans themselves via non-conservation-based approaches, such as those based on the impact of variants on protein structural stability, will be necessary to define human DDR PVs (Tam, et al., 2020).

There are limitations in our study. Although the study included 28,872 modern African individuals, the number is smaller than the ones of non-African. The unbalanced population size between African and non-African could influence the results of the PV comparison. It is well determined that African population has the most diversified genetics among global ethnic populations. However, current human genetic study is heavily biased towards European and descendent populations that contributed most of the genetic variation data currently available. This is reflected by the limited *BRCA* PV data derived from African population. The lack of genomic data from ancient African can also limit the results from the comparison. This is also the reason that we used two sets of African *BRCA* PV data in our analysis in order to compensate for the weakness. In addition, ancient human genomes are often not fully covered by sequences because of the damaged ancient DNA samples. Therefore, the actual DDR PVs in the ancient humans could be higher than identified.

In summary, by tracing the PV data in modern African, modern non-African, and ancient humans, our study demonstrates that the DDR PVs in modern humans arose mostly after the latest human out-of-Africa migration. The information provides a foundation to understand the genetic basis for disease susceptibility including cancer in modern humans.

## Data Availability

The original contributions presented in the study are included in the article/Supplementary Material, further inquiries can be directed to the corresponding author.

## References

[B1] Abkevich V. Zharkikh A. Deffenbaugh A. M. Frank D. Chen Y. Shattuck D. (2004). Analysis of missense variation in human BRCA1 in the context of interspecific sequence variation. J. Med. Genet. 41 (7), 492–507. 10.1136/jmg.2003.015867 15235020 PMC1735826

[B64] Baugh E. H. Ke H. Levine A. J. Bonneau R. A. Chan C. S. (2018). Why are there hotspot mutations in the TP53 gene in human cancers?. Cell Death Differ. 25 (1), 154–160. 10.1038/cdd.2017.180 29099487 PMC5729536

[B2] Bhaskaran S. P. Huang T. Rajendran B. K. Guo M. Luo J. Qin Z. (2021). Ethnic-specific *BRCA1/2* variation within Asia population: evidence from over 78 000 cancer and 40 000 non-cancer cases of Indian, Chinese, Korean and Japanese populations. J. Med. Genet. 58 (11), 752–759. 10.1136/jmedgenet-2020-107299 32963034

[B3] Bian L. Meng Y. Zhang M. Li D. (2019). MRE11-RAD50-NBS1 complex alterations and DNA damage response: implications for cancer treatment. Mol. Cancer 18 (1), 169. 10.1186/s12943-019-1100-5 31767017 PMC6878665

[B4] Budman J. Chu G. (2005). Processing of DNA for nonhomologous end-joining by cell-free extract. EMBO J. 24 (4), 849–860. 10.1038/sj.emboj.7600563 15692565 PMC549622

[B5] Burk-Herrick A. Scally M. Amrine-Madsen H. Stanhope M. J. Springer M. S. (2006). Natural selection and mammalian BRCA1 sequences: elucidating functionally important sites relevant to breast cancer susceptibility in humans. Mamm. Genome 17 (3), 257–270. 10.1007/s00335-005-0067-2 16518693

[B6] Cabrera V. M. (2021). Human molecular evolutionary rate, time dependency and transient polymorphism effects viewed through ancient and modern mitochondrial DNA genomes. Sci. Rep. 11, 5036. 10.1038/s41598-021-84583-1 33658608 PMC7930196

[B7] Cao Y. Li L. Xu M. Feng Z. Sun X. Lu J. (2020). The ChinaMAP analytics of deep whole genome sequences in 10,588 individuals. Cell. Res. 30 (9), 717–731. 10.1038/s41422-020-0322-9 32355288 PMC7609296

[B8] Casper M. Plotz G. Juengling B. Zeuzem S. Lammert F. Raedle J. (2012). MUTYH hotspot mutations in unselected colonoscopy patients. Colorectal Dis. 14 (5), e238–e244. 10.1111/j.1463-1318.2012.02920.x 22469480

[B9] Ceccaldi R. Sarangi P. D'Andrea A. D. (2016). The Fanconi anaemia pathway: new players and new functions. Nat. Rev. Mol. Cell. Biol. 17 (6), 337–349. 10.1038/nrm.2016.48 27145721

[B10] Chian J. S. Li J. Wang S. M. (2023). Evolutionary origin of human *PALB2* germline pathogenic variants. Int. J. Mol. Sci. 24 (14), 11343. 10.3390/ijms241411343 37511102 PMC10379391

[B11] Cline M. S. Liao R. G. Parsons M. T. Paten B. Alquaddoomi F. Antoniou A. (2018). BRCA Challenge: BRCA Exchange as a global resource for variants in BRCA1 and BRCA2. PLoS Genet. 14 (12), e1007752. 10.1371/journal.pgen.1007752 30586411 PMC6324924

[B12] David A. R. Zimmerman M. R. (2010). Cancer: an old disease, a new disease or something in between? Nat. Rev. Cancer 10 (10), 728–733. 10.1038/nrc2914 20814420

[B13] de Andrade K. C. Lee E. E. Tookmanian E. M. Kesserwan C. A. Manfredi J. J. Hatton J. N. (2022). The TP53 database: transition from the international agency for research on cancer to the US national cancer Institute. Cell. Death Differ. 29 (5), 1071–1073. 10.1038/s41418-022-00976-3 35352025 PMC9090805

[B14] de Andrade K. C. Mirabello L. Stewart D. R. Karlins E. Koster R. Wang M. (2017). Higher-than-expected population prevalence of potentially pathogenic germline TP53 variants in individuals unselected for cancer history. Hum. Mutat. 38 (12), 1723–1730. 10.1002/humu.23320 28861920 PMC6858060

[B15] Fleming M. A. Potter J. D. Ramirez C. J. Ostrander G. K. Ostrander E. A. (2003). Understanding missense mutations in the BRCA1 gene: an evolutionary approach. Proc. Natl. Acad. Sci. U. S. A. 100 (3), 1151–1156. 10.1073/pnas.0237285100 12531920 PMC298742

[B16] Freed-Pastor W. A. Prives C. (2012). Mutant p53: one name, many proteins. Genes. Dev. 26 (12), 1268–1286. 10.1101/gad.190678.112 22713868 PMC3387655

[B17] Friebel T. M. Andrulis I. L. Balmaña J. Blanco A. M. Couch F. J. Daly M. B. (2019). BRCA1 and BRCA2 pathogenic sequence variants in women of African origin or ancestry. Hum. Mutat. 40 (10), 1781–1796. 10.1002/humu.23804 31112363 PMC6764847

[B18] Fu W. Gittelman R. M. Bamshad M. J. Akey J. M. (2014). Characteristics of neutral and deleterious protein-coding variation among individuals and populations. Am. J. Hum. Genet. 95 (4), 421–436. 10.1016/j.ajhg.2014.09.006 25279984 PMC4185119

[B19] Gignoux C. R. Henn B. M. Mountain J. L. (2011). Rapid, global demographic expansions after the origins of agriculture. Proc. Natl. Acad. Sci. U. S. A. 108 (15), 6044–6049. 10.1073/pnas.0914274108 21444824 PMC3076817

[B20] Henn B. M. Cavalli-Sforza L. L. Feldman M. W. (2012). The great human expansion. Proc. Natl. Acad. Sci. U. S. A. 109 (44), 17758–17764. 10.1073/pnas.1212380109 23077256 PMC3497766

[B21] Hublin J. J. Ben-Ncer A. Bailey S. E. Freidline S. E. Neubauer S. Skinner M. M. (2017). New fossils from Jebel Irhoud, Morocco and the pan-African origin of *Homo sapiens* . Nature 546 (7657), 289–292. 10.1038/nature22336 28593953

[B22] Hudjashov G. Kivisild T. Underhill P. A. Endicott P. Sanchez J. J. Lin A. A. (2007). Revealing the prehistoric settlement of Australia by Y chromosome and mtDNA analysis. Proc. Natl. Acad. Sci. U. S. A. 104 (21), 8726–8730. 10.1073/pnas.0702928104 17496137 PMC1885570

[B23] Huttley G. A. Easteal S. Southey M. C. Tesoriero A. Giles G. G. McCredie M. R. (2000). Adaptive evolution of the tumour suppressor BRCA1 in humans and chimpanzees. Australian Breast Cancer Family Study. Nat. Genet. 25 (4), 410–413. 10.1038/78092 10932184

[B24] Jeggo P. A. Pearl L. H. Carr A. M. (2016). DNA repair, genome stability and cancer: a historical perspective. Nat. Rev. Cancer 16 (1), 35–42. 10.1038/nrc.2015.4 26667849

[B25] Jónsson H. Ginolhac A. Schubert M. Johnson P. L. Orlando L. (2013). mapDamage2.0: fast approximate Bayesian estimates of ancient DNA damage parameters. Bioinformatics 29 (13), 1682–1684. 10.1093/bioinformatics/btt193 23613487 PMC3694634

[B26] Kanehisa M. Goto S. (2000). KEGG: kyoto encyclopedia of genes and genomes. Nucleic Acids Res. 28 (1), 27–30. 10.1093/nar/28.1.27 10592173 PMC102409

[B27] Karczewski K. J. Francioli L. C. Tiao G. Cummings B. B. Alföldi J. Wang Q. (2020). The mutational constraint spectrum quantified from variation in 141,456 humans. Nature 581 (7809), 434–443. 10.1038/s41586-020-2308-7 32461654 PMC7334197

[B28] Kou S. H. Li J. Tam B. Lei H. Zhao B. Xiao F. (2023). *TP53* germline pathogenic variants in modern humans were likely originated during recent human history. Nar. Cancer 5 (3), zcad025. 10.1093/narcan/zcad025 37304756 PMC10251638

[B29] Landrum M. J. Lee J. M. Benson M. Brown G. Chao C. Chitipiralla S. (2016). ClinVar: public archive of interpretations of clinically relevant variants. Nucleic Acids Res. 44 (D1), D862–D868. 10.1093/nar/gkv1222 26582918 PMC4702865

[B30] Li H. Handsaker B. Wysoker A. Fennell T. Ruan J. Homer N. (2009). The sequence alignment/map format and SAMtools. Bioinformatics 25 (16), 2078–2079. 10.1093/bioinformatics/btp352 19505943 PMC2723002

[B31] Li J. Zhao B. Huang T. Qin Z. Wang S. M. (2022). Human *BRCA* pathogenic variants were originated during recent human history. Life Sci. Alliance 5 (5), e202101263. 10.26508/lsa.202101263 35165121 PMC8860097

[B32] Liang L. Deng L. Chen Y. Li G. C. Shao C. Tischfield J. A. (2005). Modulation of DNA end joining by nuclear proteins. J. Biol. Chem. 280 (36), 31442–31449. 10.1074/jbc.M503776200 16012167

[B33] Liu T. Huang J. (2014). Quality control of homologous recombination. Cell. Mol. Life Sci. 71 (19), 3779–3797. 10.1007/s00018-014-1649-5 24858417 PMC11114062

[B34] Lou D. I. McBee R. M. Le U. Q. Stone A. C. Wilkerson G. K. Demogines A. M. (2014). Rapid evolution of BRCA1 and BRCA2 in humans and other primates. BMC Evol. Biol. 14, 155. 10.1186/1471-2148-14-155 25011685 PMC4106182

[B35] Lynch H. T. Snyder C. L. Shaw T. G. Heinen C. D. Hitchins M. P. (2015). Milestones of lynch syndrome: 1895-2015. Nat. Rev. Cancer 15 (3), 181–194. 10.1038/nrc3878 25673086

[B36] Lynch M. (2010). Rate, molecular spectrum, and consequences of human mutation. Proc. Natl. Acad. Sci. U. S. A. 107 (3), 961–968. 10.1073/pnas.0912629107 20080596 PMC2824313

[B37] Monti P. Menichini P. Speciale A. Cutrona G. Fais F. Taiana E. (2020). Heterogeneity of *TP53* mutations and P53 protein residual function in cancer: does it matter? Front. Oncol. 10, 593383. 10.3389/fonc.2020.593383 33194757 PMC7655923

[B38] Narod S. A. Foulkes W. D. (2004). BRCA1 and BRCA2: 1994 and beyond. Nat. Rev. Cancer 4 (9), 665–676. 10.1038/nrc1431 15343273

[B39] O'Connell M. J. (2010). Selection and the cell cycle: positive Darwinian selection in a well-known DNA damage response pathway. J. Mol. Evol. 71 (5-6), 444–457. 10.1007/s00239-010-9399-y 21057781

[B40] Olivier M. Hollstein M. Hainaut P. (2010). TP53 mutations in human cancers: origins, consequences, and clinical use. Cold Spring Harb. Perspect. Biol. 2 (1), a001008. 10.1101/cshperspect.a001008 20182602 PMC2827900

[B41] Pao G. M. Zhu Q. Perez-Garcia C. G. Chou S. J. Suh H. Gage F. H. (2014). Role of BRCA1 in brain development. Proc. Natl. Acad. Sci. U. S. A. 111 (13), E1240–E1248. 10.1073/pnas.1400783111 24639535 PMC3977248

[B42] Pavlicek A. Noskov V. N. Kouprina N. Barrett J. C. Jurka J. Larionov V. (2004). Evolution of the tumor suppressor BRCA1 locus in primates: implications for cancer predisposition. Hum. Mol. Genet. 13 (22), 2737–2751. 10.1093/hmg/ddh301 15385441

[B43] Qin Z. Huang T. Guo M. Wang S. M. (2022). Distinct landscapes of deleterious variants in DNA damage repair system in ethnic human populations. Life Sci. Alliance 5 (9), e202101319. 10.26508/lsa.202101319 35595529 PMC9122833

[B44] Qin Z. Li J. Tam B. Sinha S. Zhao B. Bhaskaran S. P. (2023). Ethnic-specificity, evolution origin and deleteriousness of Asian BRCA variation revealed by over 7500 BRCA variants derived from Asian population. Int. J. Cancer 152 (6), 1159–1173. 10.1002/ijc.34359 36385461 PMC10098510

[B45] Reardon J. T. Sancar A. (2006). Purification and characterization of *Escherichia coli* and human nucleotide excision repair enzyme systems. Methods Enzymol. 408, 189–213. 10.1016/S0076-6879(06)08012-8 16793370

[B46] Richards S. Aziz N. Bale S. Bick D. Das S. Gastier-Foster J. (2015). Standards and guidelines for the interpretation of sequence variants: a joint consensus recommendation of the American College of medical genetics and genomics and the association for molecular pathology. Genet. Med. 17 (5), 405–424. 10.1038/gim.2015.30 25741868 PMC4544753

[B47] Rosen E. M. Fan S. Ma Y. (2006). BRCA1 regulation of transcription. Cancer Lett. 236 (2), 175–185. 10.1016/j.canlet.2005.04.037 15975711

[B48] Seplyarskiy V. B. Sunyaev S. (2021). The origin of human mutation in light of genomic data. Nat. Rev. Genet. 22 (10), 672–686. 10.1038/s41576-021-00376-2 34163020

[B49] Simons Y. B. Turchin M. C. Pritchard J. K. Sella G. (2014). The deleterious mutation load is insensitive to recent population history. Nat. Genet. 46 (3), 220–224. 10.1038/ng.2896 24509481 PMC3953611

[B50] Smith K. R. Hanson H. A. Hollingshaus M. S. (2013). BRCA1 and BRCA2 mutations and female fertility. Curr. Opin. Obstet. Gynecol. 25 (3), 207–213. 10.1097/GCO.0b013e32835f1731 23411475 PMC4010322

[B51] Tadaka S. Katsuoka F. Ueki M. Kojima K. Makino S. Saito S. (2019). 3.5KJPNv2: an allele frequency panel of 3552 Japanese individuals including the X chromosome. Hum. Genome Var. 6, 28. 10.1038/s41439-019-0059-5 31240104 PMC6581902

[B52] Tam B. Sinha S. Wang S. M. (2020). Combining Ramachandran plot and molecular dynamics simulation for structural-based variant classification: using *TP53*variants as model. Comput. Struct. Biotechnol. J. 18, 4033–4039. 10.1016/j.csbj.2020.11.041 33363700 PMC7744649

[B53] Tishkoff S. A. Reed F. A. Friedlaender F. R. Ehret C. Ranciaro A. Froment A. (2009). The genetic structure and history of Africans and African Americans. Science 324 (5930), 1035–1044. 10.1126/science.1172257 19407144 PMC2947357

[B54] Vallini L. Zampieri C. Shoaee M. J. Bortolini E. Marciani G. Aneli S. (2024). The Persian plateau served as hub for *Homo sapiens* after the main out of Africa dispersal. Nat. Commun. 15 (1), 1882. 10.1038/s41467-024-46161-7 38528002 PMC10963722

[B55] Volkert M. R. (1988). Adaptive response of *Escherichia coli* to alkylation damage. Environ. Mol. Mutagen 11 (2), 241–255. 10.1002/em.2850110210 3278898

[B56] Wang K. Li M. Hakonarson H. (2010). ANNOVAR: functional annotation of genetic variants from high-throughput sequencing data. Nucleic Acids Res. 38 (16), e164. 10.1093/nar/gkq603 20601685 PMC2938201

[B57] Welcsh P. L. King M. C. (2001). BRCA1 and BRCA2 and the genetics of breast and ovarian cancer. Hum. Mol. Genet. 10 (7), 705–713. 10.1093/hmg/10.7.705 11257103

[B58] Willey J. Sherwood L. Woolverton C. (2014) Prescott's microbiology. New York: McGraw Hill, 381.

[B59] Wood R. D. Mitchell M. Lindahl T. (2005). Human DNA repair genes, 2005. Mutat. Res. 577 (1-2), 275–283. 10.1016/j.mrfmmm.2005.03.007 15922366

[B60] Xiao F. Li J. Lagniton P. N. P. Kou S. H. Lei H. Tam B. (2023). Evolutionary origin of *MUTYH*Germline pathogenic variations in modern humans. Biomolecules 13 (3), 429. 10.3390/biom13030429 36979362 PMC10046817

[B61] Xue Y. Chen Y. Ayub Q. Huang N. Ball E. V. Mort M. (2012). Deleterious- and disease-allele prevalence in healthy individuals: insights from current predictions, mutation databases, and population-scale resequencing. Am. J. Hum. Genet. 91 (6), 1022–1032. 10.1016/j.ajhg.2012.10.015 23217326 PMC3516590

[B62] Zhao B. Li J. Sinha S. Qin Z. Kou S. H. Xiao F. (2024). Pathogenic variants in human DNA damage repair genes mostly arose in recent human history. BMC Cancer 24, 415. 10.1186/s12885-024-12160-6 38575974 PMC10993466

[B63] Zhivotovsky L. A. Rosenberg N. A. Feldman M. W. (2003). Features of evolution and expansion of modern humans, inferred from genomewide microsatellite markers. Am. J. Hum. Genet. 72 (5), 1171–1186. 10.1086/375120 12690579 PMC1180270

